# Clinicians’ view on non-adherence: sharing expert opinion

**DOI:** 10.3389/fphar.2025.1636806

**Published:** 2025-08-15

**Authors:** Ngiap Chuan Tan, Shashank R. Joshi, Enrique De-Madaria, Badr Eldin Mostafa, O. Nuri Özgirgin, Tommaso Simoncini, Lale Tokgözoğlu

**Affiliations:** ^1^ SingHealth Polyclinics, Singapore, Singapore; ^2^ Joshi Clinic, Mumbai, India; ^3^ Department of Gastroenterology, Dr. Balmis General University Hospital and Department of Clinical Medicine, Miguel Hernández University, Alicante, Spain; ^4^ Alicante Institute for Health and Biomedical Research (ISABIAL), Alicante, Spain; ^5^ Faculty of Medicine, Ain Shams University, Heliopolis-Cairo, Egypt; ^6^ Bayındır Sogutozu Hospital, Ankara, Türkiye; ^7^ Division of Obstetrics and Gynecology, Department of Experimental and Clinical Medicine, University of Pisa, Pisa, Italy; ^8^ Department of Cardiology, Hacettepe University Medical Faculty, Ankara, Türkiye

**Keywords:** non-adherence, medication adherence, patient compliance, adherence strategies, expert opinion

## Abstract

**Introduction:**

Medication non-adherence (NA) remains a persistent challenge across all medical specialties, contributing to adverse patient outcomes and increased healthcare burdens. While numerous studies have explored patient-related factors influencing adherence, the perspectives of healthcare professionals remain underrepresented in literature. This study aims to document the individual experiences of seven international physicians across diverse medical fields, highlighting barriers, detection methods, and strategies employed to address NA in their daily practice.

**Methodology:**

A structured qualitative approach was employed, incorporating semi-structured interviews and written questionnaires to capture expert insights. Seven physicians from specialties including family medicine, gastroenterology, otolaryngology, otology and neurotology, obstetrics and gynecology, endocrinology and cardiology participated in the study. Data were analyzed thematically to identify recurring patterns, specialty-specific challenges, and practical solutions implemented by clinicians.

**Results:**

Clinicians reported that NA detection primarily relied on patient self-reporting, clinical markers, and medication reconciliation. Barriers to adherence varied by specialty but commonly included polypharmacy, treatment complexity, patient skepticism, socioeconomic constraints, and asymptomatic conditions. Strategies to enhance adherence encompassed patient education, shared decision-making, therapeutic simplification, digital tools, and team-based care models. Despite proactive efforts, clinicians cited systemic limitations such as time constraints, fragmented healthcare records, and inadequate adherence-tracking mechanisms.

**Conclusion:**

Addressing NA requires a patient-centered, interdisciplinary approach integrating education, digital innovations, and structured follow-up strategies. The study underscores the necessity for larger-scale research to validate adherence interventions and refine multidisciplinary frameworks. Given the study’s qualitative nature and small sample size, future research should incorporate broader datasets and diverse healthcare perspectives to develop more comprehensive adherence solutions.

## 1 Introduction

Medication adherence is defined as the degree to which patients follow medical instructions. It ranges from taking their medication as prescribed to complying with diets and lifestyle changes (Brown and Bussell, 2011; Vrijens et al., 2012; Aljofan et al., 2023). The World Health Organization (WHO) categorizes adherence factors into patient, treatment, disease, socio-economic, and healthcare system-related influences (World Health Organization, 2003; Gast and Mathes, 2019; Kvarnström et al., 2021; Peh et al., 2021). Despite these insights, medication non-adherence (NA) remains a widespread challenge that affects patients across all medical specialties and care settings. NA is recognized as a multifactorial and persistent challenge across nearly all medical specialties and conditions, whether acute (e.g., malaria), chronic (e.g., hypertension), symptomatic (e.g., cystic fibrosis), or asymptomatic (e.g., dyslipidemia). Its complex causes contribute to a substantial burden on patient health, clinical practice, and the overall healthcare system (Hommel et al., 2019; Burnier et al., 2021; Lopes and Santos, 2021; Santos et al., 2022).

Although many recent studies have investigated patient adherence, the way healthcare professionals (HCPs) individually experience and address this issue varies significantly and has not yet been fully investigated in the literature (Panahi et al., 2022). The challenges they encounter are influenced by multiple factors, including healthcare setting, disease characteristics, and the individual circumstances of each patient. For example, in chronic conditions, HCPs often struggle to keep patients engaged in long-term treatment, while in acute care, the challenge may be ensuring that patients understand and follow urgent medical instructions. Beyond the medical aspects, factors like health literacy, financial constraints, and cultural beliefs about medication play a crucial role in shaping adherence.

This study aimed to document, to our knowledge for the first time in the literature, the individual perspectives of seven international physicians on medication NA in their daily practice across various medical specialties, including family medicine and primary care, gastroenterology, otolaryngology, otology and neurotology, obstetrics and gynecology (OB-GYN), endocrinology and diabetes, and cardiology. Their insights offer a nuanced understanding of how NA manifests across different fields, highlighting both common challenges and specialty-specific concerns. By examining their experiences, this study seeks to uncover the complexities of NA and explore practical strategies that HCPs can implement to enhance adherence in their respective practices, ultimately providing valuable guidance for optimizing patient adherence in routine care.

## 2 Methodology

### 2.1 Study design and interview framework

This study used a qualitative semi-structured approach to ensure a comprehensive recall of all authors’ insights and analyze their perspectives on NA. A combination of individual interviews and written questionnaires served as the primary data collection method. This dual approach preserved the authenticity of expert perspectives while capturing a diverse and well-rounded view of their clinical experiences and cultural backgrounds. Data from the semi-structured interviews and written questionnaires were collected by an independent third party to ensure objectivity.

The study design followed a multi-step process:

• Initial meeting: A preliminary meeting was held with all seven international authors to define the study objectives and key areas of interest. The experts were selected based on their interest in NA (e.g., through publications, clinical practice, or congress presentations) within their respective fields: family medicine and primary care, gastroenterology, otolaryngology, otology and neurotology, OB-GYN, endocrinology and diabetes, and cardiology.
• Preliminary data collection: Before conducting the interviews, an open-ended, free-text questionnaire was distributed via email to all authors to gather initial reflections and perspectives (a copy of the questionnaire is available in Supplementary Appendix 1).
• Development of the interview guide: Based on insights from the initial meeting and questionnaire responses, a draft interview guide was developed (a copy of the interview guide is available in Supplementary Appendix 2). The guide included open-ended questions designed to elicit in-depth responses on clinical experiences, opinions, and perspectives.
• Pilot testing: The interview guide was tested with three clinicians to assess clarity, relevance, and potential ambiguities. Revisions were made based on their feedback.
• Individual interviews: Interviews were conducted online, each lasting approximately 1 hour.

### 2.2 Data analysis

The collected data was analyzed thematically to identify common patterns and unique insights. First, the responses were synthesized into a cohesive narrative that accurately represented the collective viewpoints of the clinicians. The independent third party involved in conducting the interviews and questionnaire was also responsible for data analysis, ensuring objectivity and minimizing bias. Three individuals worked independently and simultaneously on the narrative construction and identification of key themes based on the raw data. Their reports were then shared within the group, compared, and consolidated through an iterative process to arrive at the most accurate and coherent narrative. No software was used in this analysis. This narrative was used to extract key themes and structure the manuscript, accordingly, ensuring the inclusion of all relevant perspectives. Additionally, direct quotes from physicians were incorporated to highlight individual viewpoints and provide a nuanced representation of their experiences.

Experts were informed that the meetings would be recorded and that the discussions would be used for the purposes of this manuscript. All experts agreed to these terms and provided formal consent prior to the interviews. For the questionnaire, experts were likewise informed that their responses would be used, and all provided consent to the privacy policies before proceeding.

## 3 Results: Insights from clinicians across specialties

This section explores insights from the seven physicians on NA, each offering perspectives shaped by their respective medical specialties. Their experiences highlight approaches to detecting NA, its impact on clinical practice, and the challenges associated with managing it, including specialty-specific considerations. Additionally, broader discussions address strategies to improve adherence, alongside the identified needs for enhanced training and access to robust data to support clinical decision-making.

### 3.1 Family medicine and primary care: addressing polypharmacy and aging populations

#### 3.1.1 Detection of non-adherence

Associate Professor Ngiap Chuan Tan (Singapore), specializing in family medicine, frequently encounters NA in patients with multi-morbidities. It is flagged during consultations and through pharmacist-led medication reconciliation. Discrepancies between prescribed and dispensed medications indicate adherence issues. *“Pharmacists will consult the doctors if they suspect that the patients are not taking the medication. This is an opportunity for intervention.”*

#### 3.1.2 Impact on clinical practice

NA in aging populations leads to poor health outcomes and additional physician workload. *“Patients may not fully understand the function or the purpose of taking each of the tablets,”* Prof. Tan noted, emphasizing therapeutic clarity. Limited consultation time and language barriers further complicate adherence management.

#### 3.1.3 Challenges and specialty-specific considerations

In family medicine, where continuity of care is key, NA presents unique challenges. Unlike specialists who focus on a single condition, family physicians manage a wide array of conditions simultaneously, requiring a holistic approach. Prof. Tan noted that NA in polypharmacy patients is often selective, with patients adhering to some medications while neglecting others. Furthermore, fragmented electronic health record (EHR) systems exacerbate these challenges. Limited integration between public and private HCPs hinders comprehensive tracking of patient medications and adherence. *“We do not have a clear picture of what the patients are receiving from different HCPs,”* he remarked.

### 3.2 Gastroenterology: emphasizing patient interaction and long-term monitoring

#### 3.2.1 Detection of non-adherence

Professor Enrique de Madaria (Spain), a specialist in gastroenterology with a focus on exocrine pancreatic insufficiency, emphasized the critical role of direct patient interaction in identifying NA. According to his experience, early detection often hinges on assessing the patient’s initial reaction to prescribed treatment. He highlighted that reluctance or apprehension about side effects frequently signals a higher risk of NA. “When you tell the patient the treatment you are going to start, the reaction to that information is very important to detect a risk of NA,” he noted.

Routine follow-up visits also provide opportunities to identify adherence challenges. Simple, open-ended questions such as *“Do you have problems taking the treatment?”* or *“Do you experience any issues with the medication?”* are integral to uncovering hidden barriers. Professor de Madaria stressed the importance of observing biological markers and patient-reported symptoms during follow-ups. For instance, in the context of pancreatic enzyme replacement therapy, NA may manifest unexpected symptoms such as persistent diarrhea or constipation. Such observations prompt deeper inquiries to verify whether patients are adhering to the prescribed regimen.

#### 3.2.2 Impact on clinical practice

Managing NA requires significant time investment during outpatient consultations. Professor de Madaria views this as an essential effort to ensure effective treatment outcomes. *“It’s an investment; you have to spend time, but it’s good for the physician and the patient,”* he explained. While this added responsibility increases the daily workload, it is seen as a necessary step to address the root causes of NA and improve patient care.

The long-term impact of NA varies based on the specific treatments prescribed. In the case of exocrine pancreatic insufficiency, NA may not result in immediate complications but contributes to chronic nutritional deficiencies and the potential for severe consequences over time. Professor de Madaria emphasized the importance of framing these long-term risks in discussions with patients to underline the necessity of adherence.

#### 3.2.3 Patient profiles and challenges

Professor de Madaria identified three primary patient profiles that are more likely to struggle with adherence:

• Skeptical patients: Individuals who harbor negative beliefs about medications often perceive them as harmful despite their therapeutic benefits. Such patients frequently state that medications may *“solve some issues but harm others.”*
• Patients with social or addiction issues: Those dealing with socioeconomic challenges, addiction, or unstable living conditions face unique barriers to maintaining adherence.
• Symptomatic patients blaming medications: Patients who attribute all symptoms, whether related or not, to their prescribed treatment, often express reluctance to continue the regimen.

To address these challenges, Professor de Madaria employs tailored communication strategies, emphasizing the benefits of treatment and the consequences of NA. He strives to foster a nonjudgmental environment, encouraging patients to share their genuine concerns and barriers.

### 3.3 Otolaryngology: addressing complex cases and socioeconomic barriers

#### 3.3.1 Detection of non-adherence

Professor Badr Eldin Mostafa (Egypt), a specialist in otolaryngology with a focus on head and neck malignancies, highlighted several key indicators for detecting NA in his clinical practice. These include direct questioning of patients, missed follow-up appointments, unexpected recurrence of symptoms, and, in some cases, the development of complications. He often initiates conversations about adherence by asking direct but non-confrontational questions, such as whether patients encountered difficulties finding medication or why they missed their last appointment, sometimes using a light-hearted approach to ease the dialogue.

Professor Mostafa systematically identifies non-adherent patients and has noted several at-risk profiles. These include patients with low educational status, those with very high education levels (including HCPs), individuals with low socioeconomic backgrounds, and family breadwinners who cannot afford time off work*. “The highly educated patients often delay treatment while searching for a physician who confirms their preconceived management plan,”* he noted, emphasizing how this behavior can exacerbate adherence issues.

#### 3.3.2 Impact on clinical practice

From a clinical perspective, NA significantly impacts Professor Mostafa’s day-to-day practice. It often necessitates time-consuming consultations to restart investigations and follow-ups, usually under less favorable circumstances due to disease progression. At an institutional level, NA can distort clinical data, misguide decision-making, and hinder the effective implementation of guidelines.

Professor Mostafa expressed personal frustration when dealing with non-adherent patients, especially when complaints persist or diseases progress despite available treatment options. He remarked, *“it is frustrating to restart investigations and follow-ups under less favorable circumstances due to disease progression,”* highlighting the emotional and practical toll of NA on clinicians. However, he remains vigilant and focused on early detection and proactive management to mitigate the challenges posed by NA.

#### 3.3.3 Challenges and needs in managing non-adherence

While Professor Mostafa acknowledges the universality of NA, he recognizes that its manifestations can vary by specialty. For example, in otolaryngology, adherence challenges often involve managing complex surgical and medical cases, necessitating tailored interventions. He also noted that logistical, cultural, and socioeconomic factors can significantly influence adherence patterns.

Professor Mostafa believes that addressing NA requires the involvement of adherence specialists to guide HCPs in setting up frameworks and implementing evidence-based strategies. He advocates for disease-specific studies to raise awareness among practitioners about adherence issues relevant to their specialties.

### 3.4 Otology and neurotology: addressing long-term conditions and patient motivation

#### 3.4.1 Detection of non-adherence

Professor O. Nuri Özgirgin (Turkey), an expert in otology and neurotology, focuses primarily on the treatment of chronic vestibular problems such as vertigo and equilibrium disorders. He highlighted the importance of regular follow-up visits and clinical evaluations in detecting NA. In his practice, NA often becomes evident through unexpected lab results or electrophysiological tests that reveal discrepancies in the patient’s progress. *“The follow-up process gives clues about a patient’s consistency with the treatment, providing an opportunity to directly address adherence,”* he explained.

Patients with chronic conditions that lack immediate symptoms, such as diabetes mellitus, often show higher rates of NA. However, in otology and neurotology, the earlier clinical alerts—such as worsening vertigo or balance issues—facilitate timely identification of adherence problems.

#### 3.4.2 Impact on clinical practice

NA presents significant challenges in Professor Özgirgin’s practice, especially in managing chronic vestibular conditions where adherence is crucial for effective treatment. Non-adherent patients often experience worsening symptoms, such as unsteadiness or social isolation, which require additional interventions to restore their quality of life. *“It is not easy to catch up once the breaking point has been reached. Restoring the situation comes at a financial and emotional cost for both the patient and the healthcare team,”* he noted.

Patients dealing with disabling symptoms like vertigo are generally more motivated to adhere to their prescribed treatment. However, rebuilding trust and adherence after a lapse remains a time-consuming and multifactorial process.

#### 3.4.3 Challenges and needs in managing non-adherence

While adherence is a universal issue in medicine, Professor Özgirgin pointed out that the specific challenges and interventions vary by specialty. In otology and neurotology, adherence to long-term treatments like vestibular rehabilitation or chronic dizziness therapies requires sustained effort. He noted that adherence often improves following surgical interventions, as patients anticipate short-term postoperative recovery rather than prolonged medical regimens.

He also emphasized the need for increased awareness and training among HCPs to better detect and manage NA. *“There is always something new to learn, whether it’s better detection, response strategies, or tools to intervene,”* he stated. Additionally, he advocates for scientific societies to promote adherence education through masterclasses and meeting plans.

### 3.5 Obstetrics and gynecology: overcoming fears and misconceptions

#### 3.5.1 Detection of non-adherence

Professor Tommaso Simoncini (Italy), a specialist in OB-GYN, identifies NA primarily by observing persistent symptoms despite the prescription of effective therapies. His approach includes direct inquiries with patients about potential challenges they faced with the treatment, including inconvenience, lack of perceived benefit, or fears about side effects. Given the frequent use of hormonal therapies in his field, he pays particular attention to whether patients are influenced by external advice or concerns about potential risks such as weight gain or cancer.

Although Professor Simoncini does not systematically identify NA, he becomes vigilant when he perceives resistance or skepticism from patients. Certain patient profiles are particularly challenging, including those with preconceived doubts about treatment and heightened fears about side effects.

#### 3.5.2 Impact on clinical practice

From a clinical perspective, NA significantly impacts Professor Simoncini’s practice by contributing to the chronicization of conditions that could otherwise be resolved. Over time, these conditions become less treatable, representing a lost opportunity for effective care. He observed that re-initiating treatment after prolonged NA often yields diminished results despite intensive efforts to educate and reassure patients.

For Professor Simoncini, addressing NA requires strong communication skills to help patients understand the consequences of NA. He emphasized the frustration of not being able to effectively convey reliable messages to patients, as it undermines their trust and engagement with the prescribed therapy.

#### 3.5.3 Challenges and needs in managing non-adherence

Professor Simoncini highlighted the pervasive challenge of miscommunication in OB-GYN. He noted that lingering fears and misconceptions about common treatments—ranging from contraception to menopause management—undermine adherence across various subspecialties. Addressing these challenges requires targeted education and evidence-based resources.

He expressed a need for structured strategies and materials to share with patients, such as physical handouts or digital aids that explain the importance of adherence and its consequences. Additionally, he called for more scientific studies documenting the impact of NA in OB-GYN to strengthen the evidence base for patient education.

### 3.6 Endocrinology and diabetes: managing chronic conditions and behavioral factors

#### 3.6.1 Detection of non-adherence

Professor Shashank R. Joshi (India), an endocrinologist and diabetologist, identifies NA through a combination of patient, caregiver, and healthcare team feedback. Patients often disclose their NA out of guilt, or caregivers report it during consultations. Additionally, healthcare assistants, such as diabetes nurses or educators, may flag inconsistencies when patient records indicate suboptimal outcomes.

Professor Joshi systematically addresses adherence during each consultation, ensuring that all patients are directly questioned about their medication, diet, and exercise adherence. He uses structured questionnaires, administered by HCPs, to document adherence patterns. While laboratory tests are occasionally used to suspect NA, their application is limited to clinical trials or specific contexts.

In Professor Joshi’s practice, certain patient profiles are more prone to NA, including those with addictive behaviors (e.g., smokers or alcohol users), individuals who are overly reliant on lifestyle modifications, and patients experiencing economic hardships. Interestingly, highly committed lifestyle adherents may neglect prescribed medications, believing that lifestyle changes alone suffice. *“We have observed a peculiar phenotype where patients committed to lifestyle changes sometimes neglect their medications, believing they can cure their diabetes solely through lifestyle modifications.”*

#### 3.6.2 Impact on clinical practice

NA significantly impacts Professor Joshi’s clinical workload, with approximately 30% of his patients exhibiting adherence issues. In his opinion, managing these patients requires 25% more consultation time compared to adherent patients. This increased burden extends to his healthcare team, particularly his assistants and nurses, who are actively involved in identifying and addressing NA.

The repercussions of NA include complications, worsened conditions, and additional healthcare interventions. This creates a vicious cycle, increasing both patient hardships and the workload of the caregiving team. From a personal perspective, Professor Joshi has evolved from feeling frustrated and agitated by NA to adopting a more constructive approach focused on addressing its underlying causes and implementing proactive solutions.

#### 3.6.3 Challenges and specialty-specific considerations

In endocrinology, NA often arises due to the asymptomatic nature of chronic conditions like diabetes and thyroid disorders. Patients may stop medications once biological markers normalize, failing to recognize the long-term necessity of treatment. Professor Joshi emphasizes the importance of measurable outcomes, such as blood sugar levels or thyroid markers, as motivators for adherence.

Despite the measurable benefits of adherence, chronic care specialties face unique challenges compared to acute care, where adherence is often higher due to immediate supervision. The long-term, unsupervised nature of chronic disease management requires more persistent efforts to engage patients and ensure adherence.

### 3.7 Cardiology: managing chronic disease and long-term commitment

#### 3.7.1 Detection of non-adherence

Professor Lale Tokgözoğlu (Turkey), an experienced cardiologist, highlights that the detection of NA in her practice is primarily facilitated by clinical markers. In cardiology, expected improvements in blood pressure, lipid levels, and other biomarkers typically serve as clear indicators of adherence. When these markers fail to improve as anticipated, it raises suspicion of NA. *“The likelihood of being refractory to a medicine is extremely low,”* she states, emphasizing that deviations are often a result of missed doses or incomplete adherence rather than therapeutic ineffectiveness.

Initiating a conversation about adherence is approached delicately and without blame. Professor Tokgözoğlu explains, *“I systematically say, ‘You are taking this regularly, right?’”* before proceeding to further discussion. This gentle inquiry often leads patients to admit to lapses in adherence, such as forgetting doses or failing to refill prescriptions. By framing the issue as a shared problem and discussing potential solutions, patients feel less defensive and more willing to disclose.

Patients more prone to NA include those who exhibit reluctance toward lifelong medications, individuals with polypharmacy, or those influenced by misinformation—a growing challenge in the age of social media. Additionally, younger patients who question the need for long-term treatments and older adults facing challenges with regimen complexity are at higher risk.

#### 3.7.2 Impact on clinical practice

NA presents a significant burden on Professor Tokgözoğlu’s clinical practice. Addressing NA requires additional time and effort, particularly for shared decision-making and patient education. She notes, *“it certainly needs more time and more convincing,”* as it often involves understanding patient concerns, managing potential side effects, and tailoring interventions.

The consequences of NA are often severe and lead to complications such as strokes, ventricular hypertrophy, or elevated blood pressure. These complications not only affect patient health outcomes but also increase the complexity of subsequent medical management. Despite these challenges, Professor Tokgözoğlu remains pragmatic: “I feel it’s my duty to align them with scientific facts,” she explains, emphasizing the importance of providing evidence-based guidance amidst widespread misinformation.

#### 3.7.3 Challenges and specialty-specific considerations

Professor Tokgözoğlu underscores that the challenges of NA in cardiology are influenced by the asymptomatic nature of many conditions. For instance, patients may not perceive immediate benefits from taking statins, as high cholesterol does not present obvious symptoms. She highlights, *“When you do not take your cholesterol medication, nothing happens,”* making it difficult to sustain adherence. In contrast, the acute symptoms of other conditions, such as hypertension-related headaches, may serve as a natural motivator for adherence.

Additionally, she notes that the effectiveness of adherence strategies varies based on individual patient profiles. Educational materials, whether print or digital, must be adapted to the patient’s age, literacy level, and access to technology.

### 3.8 Needs for training and data

Some of the interviewed physicians emphasized the need for enhanced training and data-driven approaches to optimize the management of medication NA. A unified national EHR system was identified as crucial for tracking prescriptions and dispensed medications across healthcare providers, improving coordination and adherence monitoring. Digital solutions, including mobile applications, AI-driven risk assessments, and smart pillboxes, were highlighted as promising tools, particularly for elderly patients with cognitive challenges. However, effective integration of these technologies requires standardized training for HCPs to ensure their appropriate use.

In addition to technological advancements, the need for team-based care models was underscored, advocating for the active involvement of pharmacists, nurses, and administrative staff in adherence management. Training programs should focus on equipping HCPs with skills to detect and address NA, incorporating motivational techniques and behavioral strategies. Furthermore, generating robust scientific data on the clinical consequences of NA is essential to raise awareness and drive systemic improvements. Time constraints, particularly in high-volume clinical settings, were recognized as a major challenge, reinforcing the need for structured training programs, particularly for younger clinicians. Providing guidance on evidence-based digital tools would further support clinicians in integrating technology effectively into patient care. A multidisciplinary, data-driven, and technology-enhanced approach was recommended to strengthen adherence management strategies.

### 3.9 Strategies to improve adherence

The seven physicians interviewed outlined a range of strategies to enhance medication adherence, tailored to their respective specialties and patient populations. Common themes emerged across their approaches, emphasizing patient education, behavioral interventions, and system-level improvements. Shared decision-making and proactive communication were widely endorsed, ensuring that patients understand their conditions, treatment benefits, and potential consequences of NA. Many physicians employed tailored regimens, deprescribing where possible, and leveraging behavioral techniques such as linking medication intake to daily routines. Practical tools, including pill organizers and digital reminders, were frequently recommended, though their suitability varied by patient demographics, particularly among older populations. Several physicians highlighted the importance of multidisciplinary involvement, integrating pharmacists, nurses, and social workers to reinforce adherence strategies. Economic and logistical barriers were also addressed through customized solutions, including financial assistance programs and simplified treatment regimens. Additionally, therapeutic education, both in clinical settings and through public awareness campaigns, was recognized as a critical component in fostering long-term adherence. While digital solutions, such as adherence-tracking applications, were identified as promising, their effectiveness remained contingent on patient familiarity with technology. Overall, a multifaceted, patient-centered approach—combining education, behavioral reinforcement, tailored interventions, and multidisciplinary support—was advocated to optimize adherence outcomes.

## 4 Discussion

The findings of this study illustrate the complexity of NA, its diverse manifestations, and the strategies clinicians employ to mitigate its impact across different specialties. Key barriers to adherence include patient-related factors such as cognitive decline, skepticism, and socioeconomic constraints, alongside disease and treatment-related challenges like polypharmacy, regimen complexity, and asymptomatic conditions. Healthcare system inefficiencies, including fragmented electronic health records and limited consultation time, further complicate adherence management.

A recent study by the European Network to Advance Best Practices and Technology on Medication Adherence (ENABLE) identified major challenges in NA, including low patient awareness, insufficient time for HCPs, inadequate digital solutions, and poor interprofessional collaboration (Hafez et al., 2024). While these systemic issues are significant, they do not fully encompass the multifaceted nature of NA, particularly within the clinical contexts explored in our study. Although ENABLE advocates for enhanced education and digital interventions, our findings emphasize the need for individualized, patient-centered approaches. NA is often driven by specific patient profiles—such as individuals skeptical of medications or those facing complex social challenges—necessitating tailored interventions. This underscores the limitations of purely technological solutions and highlights the importance of culturally aware, context-sensitive care strategies to improve adherence outcomes.

HCPs employ various strategies to assess the risk of NA upon a first consultation. Beginning with simple inquiries, they identify at-risk groups and adherence barriers. Interviewing patients about adherence is the most used method despite its low reliability, as it relies on the patient’s honesty and is subjected to the white coat effect (Hamrahian et al., 2022; Burnier, 2024). Observing patients' reactions to discussions about new treatments is key, especially if the treatment is long term; reluctance may signify potential NA. To prevent NA, thorough explanations of the disease and the prescribed treatments are essential. Unfortunately, the physician’s time is limited during a consultation, with only about 5 minutes allocated to discussing treatment adherence (Burnier, 2024). However, during follow-ups, detection of NA often relies on voluntary disclosures from patients or caregivers, direct questioning, inquiring about the patients’ current satisfaction with the treatment. Nonetheless, not all physicians investigate NA systematically, some of them rely on their connection with the patient to assess NA and inquire only when they feel it necessary. Sometimes, laboratory analysis could be more reliable for doctors to assess their patient’s adherence whether it is by detection of the compound or through biological markers.

While biological markers are not definitive indicators in every specialty, lack of medication efficacy can suggest NA and more specifically in asymptomatic conditions. For instance, the use of statins should result in a decrease in the patient’s blood cholesterol. If the low-density lipoprotein (LDL) cholesterol levels remain identical, NA should be investigated (Lansberg et al., 2018). The same principle can be applied to antihypertensive medication with the blood pressure measure. Some comorbidities such as dementia, anxiety, or diabetes can also lead to lower adherence whereas hypertension is associated to a higher adherence to lipid lowering drugs. These identifiable factors can help physicians tailor their approach when facing potentially non-adherent patients (Lopes and Santos, 2021).

Persisting symptoms or complications, missed appointments, and unexpected return of symptoms also flag potential NA. The conversation with patients, initiated with sensitivity, should balance direct questions with gentle questioning into adherence barriers including medication cost, management of side effects and the psychological impact of a lifetime treatment. Using a valid, reliable, cost-effective, straightforward, and readily accessible objective method would be the gold standard in NA detection. However, simpler and less expensive methods often come with lower reliability. In contrast, methods with higher reliability tend to be more expensive and require more infrastructure (Hamrahian et al., 2022). HCPs are forced to rely on clues given by their patients to identify those at risk of poor adherence. Recognizing these profiles and employing tailored approaches can enhance adherence and optimize patient outcomes.

Clinicians often find themselves allocating considerable extra time to address the needs of non-adherent patients, which can amount to a 25% increase compared to adherent peers. Non-adherent patients typically need three extra consultations annually compared to their adherent counterparts (Cutler et al., 2018). This investment is not merely a matter of convenience but a critical component of effective patient care; neglecting it risks exacerbating patients' conditions and complicating treatment pathways. In cardiology alone, poor adherence to cardiovascular medication is directly linked to an increase in cardiovascular events and mortality. An improvement of 20% in adherence is associated with 140 fewer deaths from all-causes per 1 million per year (Chen et al., 2022). This highlights the significant role of the clinicians taking the time to address NA.

The consequences of NA ripple through the healthcare system, leading to worsening conditions, increased reliance on medication, and a shift from manageable to chronic illnesses. Despite the hidden nature of some immediate consequences, the long-term impacts are palpable, both in terms of patient outcomes and the strain placed on healthcare providers. Addressing NA requires not only clinical acumen but also patience and persistence in conveying the importance of treatment compliance. Failure to address this issue not only undermines the quality of care but also represents a missed opportunity to alleviate future complications and enhance patient wellbeing.

## 5 Limitations

The study is limited by its qualitative nature and the relatively small sample size, which may not fully capture the perspectives across the different clinical settings and specialties, thereby limiting the generalizability of our findings. While the findings provide valuable insights into clinicians' individual views on medication NA future research should aim to incorporate larger datasets, potentially through broader surveys, to provide a more comprehensive and representative understanding of the factors influencing NA. Expanding the scope of investigation to include additional HCPs and patient perspectives could also enrich the findings and contribute to a more holistic view of adherence challenges and potential solutions.

## 6 Conclusion

Medication NA is a widespread challenge requiring patient-centered, tailored interventions to improve outcomes. The insights from clinicians emphasize the critical role of personalized strategies in detecting and addressing adherence issues. By prioritizing tailored communication, regular follow-ups, and a deeper understanding of individual patient challenges, clinicians can more effectively manage NA. While systemic barriers such as limited patient awareness, time constraints for HCPs, and technological limitations persist, our findings suggest that a flexible, individualized approach is most effective. A team-based model that integrates direct patient-clinician interaction with systemic support and digital innovations holds promise for enhancing adherence and improving patient outcomes. Future research should prioritize validating digital adherence tools, exploring psychological determinants of NA, assessing the impact of multidisciplinary care models, and investigating policy-level changes to enhance adherence support.

## Data Availability

The original contributions presented in the study are included in the article/Supplementary Material, further inquiries can be directed to the corresponding author.

## Abbreviations

EHR, Electronic health record; ENABLE, European Network to Advance Best Practices and Technology on Medication Adherence; HCPs, Healthcare professionals; LDL, Low-density lipoprotein; NA, Non-adherence; OB-GYN, Obstetrics and gynecology; WHO, World Health Organization.
