# Supplementary material for: Clinicians’ view on non-adherence: sharing expert opinion
Source: Front Pharmacol. 2025 Aug 15;16:1636806. doi: 10.3389/fphar.2025.1636806 (PMC12396120; doi:10.3389/fphar.2025.1636806)
Supplement: Supplementary file 1 [file Supplementaryfile1.docx]

**FRONTIERS IN PHARMACOLOGY: Special issue on Medication Adherence**

**Paper: Clinicians' view on non-adherence: sharing expert opinion**

**Written questionnaire guide**

This questionnaire was designed to gather insights from clinical experts regarding their experiences and perspectives on medication non-adherence (NA). The responses will contribute to a qualitative manuscript capturing clinicians’ day-to-day experiences and incorporating direct quotations to illustrate key themes.

**Objective of the Manuscript**

- The manuscript aims to reflect clinicians’ real-world experiences with medication NA in their daily practice.
- It follows a qualitative approach, differing from strictly research-based papers.

**Clinician Questionnaire**

Please answer the following questions in detail and provide background or explanatory information to support your responses.

**1. Detection of NA**

1.1 How do you typically detect or recognize medication non-adherence in your patients?
1.2 How do you approach and initiate conversations about non-adherence with your patients?
1.3 Do you systematically assess non-adherence in your practice? If so, how?
1.4 Are there specific patient profiles or characteristics that present greater challenges in terms of adherence?

**2. Impact of Non-Adherence on Clinical Practice**

2.1 From your perspective, how does medication non-adherence impact your daily clinical practice?
2.2 What additional effort or adjustments are required from you to address non-adherence effectively?
2.3 Do non-adherent patients often return with:

- Worsening of their condition?
- New or exacerbated complications?
- Additional burdens that affect your clinical workload?
  2.4 How does managing non-adherence affect you personally and professionally?
- How do you perceive your role in helping these patients?
- What emotions or challenges do you experience in this context?

**3. Clinician Interventions to Improve Adherence**

3.1 What strategies do you use in clinical practice to improve adherence?
3.2 Are your adherence interventions general or tailored to specific patient profiles?
3.3 Do you implement adherence interventions personally, or do you refer patients to other healthcare professionals (e.g., nurses, pharmacists)?
3.4 What tools do you use to support adherence (e.g., digital tools, adherence monitoring systems)?

**4. Clinicians’ Needs Regarding NA Management**

4.1 Do you feel you need additional training in any of the following areas?

- Understanding the causes of non-adherence?
- Detecting non-adherence more effectively?
- Communicating with and supporting non-adherent patients?
- Utilizing available intervention tools (digital and non-digital)?
  4.2 Do you believe additional scientific studies are needed to better document the consequences of non-adherence for specific conditions or treatments?

**5. Challenges and Specialty-Specific Considerations in NA**

5.1 Are there specific challenges related to non-adherence that are unique to your specialty or practice?
5.2 In your opinion, is non-adherence a universal issue, or does it require specialty-specific approaches to management?
5.3 Do you believe adherence management should involve specialists within each discipline, or can it be addressed through general clinical strategies?
5.4 Would having dedicated adherence specialists be beneficial for healthcare professional education on this issue?
