# Supplementary material for: Clinicians’ view on non-adherence: sharing expert opinion
Source: Front Pharmacol. 2025 Aug 15;16:1636806. doi: 10.3389/fphar.2025.1636806 (PMC12396120; doi:10.3389/fphar.2025.1636806)
Supplement: Supplementary file 2 [file Supplementaryfile2.docx]

**FRONTIERS IN PHARMACOLOGY: Special issue on Medication Adherence**

**Paper: Clinicians' view on non-adherence: sharing expert opinion**

**Interview guide**

This guide was used to explore clinicians’ perspectives on medication non-adherence (NA), including its detection, impact, interventions, and challenges.

**1. Detection of NA**

1.1 How do you typically identify or detect medication non-adherence in your patients?
1.2 How do you initiate conversations about non-adherence with your patients?
1.3 Are there specific patient profiles or characteristics that you find more challenging in terms of adherence?

**2. Impact of NA on Clinical Practice**

2.1 From your perspective as a clinician, how does non-adherence affect your daily clinical practice?
2.2 Among non-adherent patients, do you often observe:

- Worsening of their condition?
- Increased complications?
- Development of new health issues?
  2.3 How does dealing with non-adherence impact you personally and professionally?
- What emotions or challenges do you experience when managing non-adherent patients?
- How do you perceive your role in supporting these patients?

**3. Clinician Interventions for Improving Adherence**

3.1 What strategies or approaches do you use in clinical practice to improve medication adherence?
3.2 Do you utilize specific tools to support adherence (e.g., digital tools, adherence monitoring systems)?
3.3 Do you personally implement adherence interventions, or do you refer patients to other healthcare professionals (e.g., nurses, pharmacists)?

**4. Clinicians’ Needs Regarding NA Management**

4.1 Do you feel you require additional training in any of the following areas?

- Understanding the root causes of non-adherence?
- Detecting non-adherence more effectively?
- Communicating with and responding to non-adherent patients?
- Using available tools (digital or non-digital) to support adherence?
  4.2 Are there any other needs or resources that could help you address non-adherence more effectively?

**5. General and Specific Challenges Related to Non-Adherence**

5.1 What are the key challenges you face when addressing medication non-adherence?
5.2 Are there any specific barriers—clinical, systemic, or patient-related—that make it difficult to improve adherence?
